# Peer Presence Effects on Eye Movements and Attentional Performance

**DOI:** 10.3389/fnbeh.2019.00280

**Published:** 2020-01-08

**Authors:** Leslie Tricoche, Johan Ferrand-Verdejo, Denis Pélisson, Martine Meunier

**Affiliations:** INSERM, U1028, CNRS, UMR5292, Lyon Neuroscience Research Center, ImpAct Team, University Lyon, Bron, France

**Keywords:** social facilitation, social cognition, social presence, oculomotor behavior, saccades, LATER model, attention

## Abstract

“Social facilitation” refers to the enhancement or impairment of performance engendered by the mere presence of others. It has been demonstrated for a diversity of behaviors. This study assessed whether it also concerns attention and eye movements and if yes, which decision-making mechanisms it affects. Human volunteers were tested in three different tasks (saccades, visual search, and continuous performance) either alone or in the presence of a familiar peer. The results failed to reveal any significant peer influence on the visual search and continuous performance tasks. For saccades, by contrast, they showed a negative or positive peer influence depending on the complexity of the testing protocol. Pro-and anti-saccades were both inhibited when pseudorandomly mixed, and both facilitated when performed separately. Peer presence impaired or improved reaction times, i.e., the speed to initiate the saccade, as well as peak velocity, i.e., the driving force moving the eye toward the target. Effect sizes were large, with Cohen’s *d*-values ranging for reaction times (RTs) from 0.50 to 0.95. Analyzing RT distributions using the LATER (Linear Approach to Threshold with Ergodic Rate) model revealed that social inhibition of pro- and anti-saccades in the complex protocol was associated with a significant increase in the rate of rise. The present demonstration that the simple presence of a familiar peer can inhibit or facilitate saccades depending on task difficulty strengthens a growing body of evidence showing social modulations of eye movements and attention processes. The present lack of effect on visual search and continuous performance tasks contrasts with peer presence effects reported earlier using similar tasks, and future studies are needed to determine whether it is due to an intermediate level of difficulty maximizing individual variability. Together with an earlier study of the social inhibition of anti-saccades also using the LATER model, which showed an increase of the threshold, the present increase of the rate of rise suggests that peer presence can influence both the top-down and bottom-up attention-related processes guiding the decision to move the eyes.

## Introduction

### Social Presence Effects on Performance

Social psychology has long established that others’ presence influences individuals’ behaviors. The first evidence can be traced back to an 1898 study by Triplett (1898). In Allport (1924) named this phenomenon “social facilitation.” Later, Zajonc (1965) established that others’ presence actually facilitates only well-learned responses, novel responses being, on the contrary, inhibited. Allport’s early label nonetheless stuck and, to this day, “social facilitation” refers to any *enhancement* or *impairment* of performance due to the presence of others (Monfardini et al., 2017). A century of social psychology has built a robust knowledge about the principles ruling this fundamental form of social influence. The main ones are that others influence behavior whether their presence is actual or imagined, whether they are familiar or unknown, co-actors doing the same task or passive spectators, and whether they are evaluative or neutral (Bond and Titus, 1983; Guerin, 2010; Reynaud et al., 2015). Two issues remain unsolved though: first, the mechanism mediating social facilitation and second, the factors which make an individual more or less susceptible to others’ presence. These unsolved issues represent a limitation, making it difficult to translate laboratory findings about social facilitation into real-life applications to domains, such as school or work, where others are omnipresent.

### Social Facilitation Mechanism

Among the many theories proposed by social psychology, two prominent ones put the spotlight on attention processes. Zajonc (1965) proposed arousal, a vigilance mechanism as social facilitation mediator, whereas Baron proposed distraction, a selective attention mechanism (Baron, 1986). The two theories being not mutually exclusive, an attention theory positing that others’ presence acts *via* both vigilance and selective attention is a plausible alternative (Huguet et al., 2014; Monfardini et al., 2017). Attention in all primates, humans included, rely mostly on vision. Our visual environment containing more information than can be processed simultaneously, it is necessary to select the behaviorally most relevant information for further processing and to filter out the unwanted information. This vital ability is known as selective attention and is tightly linked with eye movements; the two functions sharing the same frontoparietal network in the brain (Corbetta et al., 1998). Selective attention and its underlying frontoparietal network are themselves modulated by the locus coeruleus noradrenergic neurons, which are known to play an important role in vigilance, the ability to sustain attention to a task for a long period of time (Sara, 2009). Neuroscience-based evidence could, therefore, complement social psychology behavioral findings and help specify the attention mechanisms at play in social facilitation and tease them apart from other possible mediators such as motivation (Harkins, 2006).

To date, much of the research effort of neuroimaging studies of social facilitation has focused on pleasurable behaviors in adolescents and adults, such as gaming for money (Nawa et al., 2008; Fareri et al., 2012; Kätsyri et al., 2013; Breiner et al., 2018; Chib et al., 2018), donating to charities (Izuma et al., 2010; Van Hoorn et al., 2016) and risk-taking (Chein et al., 2011; Smith et al., 2015, 2018; Hoffmann et al., 2018). In these studies, peer presence’s most consistent effect was an increase of activation or connectivity in the brain reward system, especially the ventral striatum. This striatal increase is proportional to participants’ subjective enhancement of pleasure (Kätsyri et al., 2013). Of interest to the present study, which investigates familiar peers, friends yield greater pleasure and greater striatal activation than strangers (Fareri et al., 2012). As yet, few neuroimaging studies have addressed peer presence effects on sensorimotor or cognitive behaviors rather than hedonic activities. In a princeps study, we described increased activation in the frontoparietal attention network during socially facilitated image pressing in monkeys (Monfardini et al., 2016). The link between social facilitation and the brain parietal areas involved in attention has now been corroborated by at least three human studies, one using a motor task (Yoshie et al., 2016), one testing sensory judgments (Müller-Pinzler et al., 2015), and one assessing cognitive reasoning (Dumontheil et al., 2016). Together, these findings provide neural evidence in support of the attentional theories of social facilitation proposed by social psychology.

### Social Attention Literature

Research on social attention, i.e., on how information about other people affects attentional processes, has long suggested a special status of social cues among environment cues. Much of the effort focused on others’ gaze direction and its impact on spatial orienting. It showed that other’s gaze direction can reallocate our attention not always in an automatic, bottom-up way, determined by its sole physical saliency, but also in a voluntary, top-down way, driven by both the observer’s social relevance and the subject’s goals (Koval et al., 2005; Birmingham and Kingstone, 2009; Greene et al., 2009; Chauhan et al., 2017; Atkinson et al., 2018). In this eye gaze domain too, familiar peers may have special effects (Chauhan et al., 2017).

By contrast, how others’ mere presence affects attention processes, and the eye movements upon which they rest has long been undocumented. Only over the last 3 years can we found behavioral studies investigating social presence effects in visual search tasks assessing selective spatial attention (Yu and Wu, 2015; Liu and Yu, 2017), in a continuous performance task (CPT) assessing vigilance, i.e., attention sustained over time (Claypoole and Szalma, 2017), and in saccades tasks assessing eye movements, both pro- and anti-saccades (McFall et al., 2009; Strukelj et al., 2012; Oliva et al., 2017). The data remain patchy and are sometimes contradictory, but they provide the proof of concept that all three facets of attention (eye movements, attention in space, and attention in time) can be modified by others’ mere presence. To assess underlying mechanisms, one study (Oliva et al., 2017) complemented behavior with computational modeling, fitting eye movement data to Carpenter’s LATER (Linear Approach to Threshold with Ergodic Rate) model, which postulates two different mechanisms leading to the decision to act, i.e., move the eye or hand: top-down decision threshold and bottom-up accumulation of information (Carpenter and Williams, 1995; Carpenter, 2012).

The present study participates in this recent endeavor. We use the same three tasks as in the above studies, namely, pro-/anti-saccades, visual search, and CPT. All three are well-established tools in the attention literature to assess the oculomotor, spatial, and temporal facets of attention, respectively, and our lab has extensive experience with them (Gerardin et al., 2015; Habchi et al., 2015; Nicolas et al., 2019). The difference with earlier studies is that, first, all three tasks are tested in the same group of subjects, rather than across different groups, and second, social presence is embodied by a familiar peer rather than by a stranger or a group of strangers. We chose familiar peers based on evidence that they are more effective social facilitation triggers than strangers. This includes the evidence from the attention literature evoked above (Fareri et al., 2012; Chauhan et al., 2017), plus similar evidence from primatology (Wechkin, 1970) and social psychology (Herman, 2015). We also chose familiar peers because of their omnipresence at school or work, the two daily life situations that could benefit most from novel findings in social facilitation research.

### Objectives of Present Study

In summary, the present study assesses the influence of the mere presence of a familiar peer on pro-/anti-saccades, visual search, and CPT, i.e., eye movements, attention in space, and attention in time, respectively. When applicable (for saccades and CPT), behavioral analyses of eye and hand reaction times were completed by computational analyses using Carpenter’s LATER model. To evaluate the moderating effect of difficulty, each task pseudorandomly mixed two types of trials. Natural pro-saccades were mixed with atypical anti-saccades, pop-out targets with hard-to-find targets in visual search, and frequent NoGo responses with rare Go responses in CPT. Based on Zajonc (1965), we initially expected, for each task, social facilitation of easy responses and social inhibition of challenging ones. As collected data showed no such within-task difference, yielding instead a social inhibition of both pro- and anti-saccades, the second group of subjects was recruited to test difficulty across rather than within tasks. This second group performed pro- and anti-saccades successively, a simpler task than the mix of pro- and anti-saccades administered to the first group, which makes natural pro-saccades much more difficult (Pierce and McDowell, 2016, 2017).

## Materials and Methods

### Ethics

The Ethics Committee of Inserm (IRB#00003888) approved the study (November 3, 2015) which was conducted according to the principles expressed in the Declaration of Helsinki. All participants provided written informed consent before performing the tasks and received compensation for their participation. All methods were performed in accordance with the relevant guidelines and regulations.

### Participants

A total of 79 university students were recruited either directly in the laboratory, or *via* web posting (47 females, 57%, mean age 23.2 years, SD = 3.2, range: 19–35 years, with normal or corrected vision and no neurologic or psychiatric history). They were told they could come alone or with a same-age familiar partner of their choice (colleague, friend, sibling, or lover). No reason was given as to why duos were welcome to participate. Eleven socially tested duos (i.e., a total of 22 subjects; Social condition) and 21 individually tested subjects (Alone condition) successively performed, in a single session, the complex version of the saccades task (mixed pro- and anti-saccades), the visual search task, and the CPT, in that order. The remaining 36 subjects (nine duos i.e., 18 subjects in the Social condition, and 18 subjects in the Alone condition) solely performed the simple version of the saccades task (successive pro- and anti-saccades).

### Familiarity Assessment

The IOS, Inclusion of Other in the Self-scale (Gächter et al., 2015) was used to ascertain familiarity within the duos along a 7-point scale (1 = “not close at all,” 7 = “very close”). Only the subjects with IOS scores ≥4, reflecting close relationships (Aron et al., 1997; Myers and Hodges, 2012), were retained in the analyses.

### Set-Up: Social vs. Alone Condition

**Figure 1:**
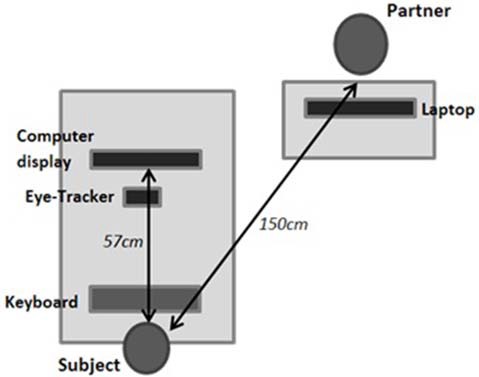
Experimental setup. In the social condition, the partner is located in front and to the right of the subject.

While performing the tasks, the subject was seated in an adjustable chair, facing the eye-tracker camera (“EyeLink1000” SR-Research) and computer screen with the head on a chin rest and forehead support, the eyes aligned with the screen center, and the hands over a computer keyboard (Figure 1). For the Social condition, the familiar partner completed questionnaires on a laptop while seated on the subject’s right side without any possibility of judging or even seeing the subject’s performance; instructions related to the tasks and questionnaires were given to both individuals at the same time, just after entering the testing room. The experimenter left the room immediately after launching the appropriate computer program. The subject used a walkie-talkie to signal the end of the session to the experimenter who, for duos, then came back and reversed the roles, the familiar partner becoming the actor and vice versa.

### Tasks

The tasks were developed using the EyeLink^®^ Experiment Builder software.

#### Saccades Task

**Figure 2:**
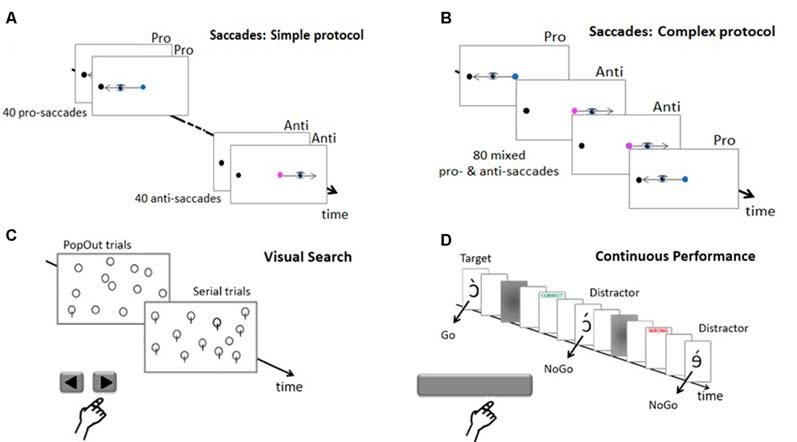
Tasks. **(A)** Saccades trials in the simple protocol and **(B)** in the complex protocol. As soon as the peripheral target appeared, subjects must look at it (pro-saccade) if the cue is blue or at the opposite location (anti-saccade) if the cue is pink. **(C)** Visual Search Pop Out and Serial trials. Subjects must report whether a target (a plain circle or a circle with a bar against distractors of the other category) was present or not by pressing the right or left arrow key. **(D)** Continuous performance task (CPT). The subject must report targets by releasing the space-bar key and refrain from answering when distractors are presented. Informative feedback about precision and speed was provided in all three tasks.

Oculomotor behavior was assessed using both pro-saccade (saccades toward the target) and anti-saccades (saccades away from the target, toward its mirror location). Subjects were asked to fixate a dot cue on the screen center (Figures 2A,B). After a randomized time-period (1,000, 1,500, 2,000, 2,500, 3,000 or 3,500 ms) a black dot target was flashed for 30 ms at 15° on the horizontal axis in the right or the left visual hemifield. If the cue was blue, subjects had to produce a pro-saccade, whereas if it was pink, they had to produce an anti-saccade. The cue remained visible until after the execution of the saccade (overlap paradigm). In all trials, subjects had to respond as fast and precisely as possible, and an informative “TOO SLOW” feedback appeared on the screen if saccade latency exceeded 510 ms. The cue then disappeared, leaving a blank screen for 200 ms before the next trial began. Subjects performed 40 pro-saccades and 40 anti-saccades. Two protocols were used (in different subjects). In the simple protocol (Figure 2A), pro-saccades and anti-saccades were presented successively in two separate blocks of 40 trials each. In the complex protocol (Figure 2B), pro-saccades and anti-saccades were mixed, appearing pseudo-randomly in a single block of 80 trials, and could thus not be anticipated by the subjects before the cue appearance. All subjects performed 10 training trials before the task. The number of experimental trials measured per saccade type in each protocol (one block of 40) was thus lower than the number (three blocks of 40) recommended by Antoniades et al. (2013) in their 2013 proposal for a standardized pro-/anti-saccade protocol, but this reduction was necessary in our case to make it possible to test two attention tasks in addition to saccades.

#### Visual Search Task

Selective attention in space was assessed using a visual search task pseudo-randomly mixing easy detection of salient, “pop-out” targets, and lengthy “serial” searches for hard-to-detect targets (Khan et al., 2016). Subjects had to find a target among distractors (Figure 2C). This visual scene was preceded by a central fixation point presented for a randomized period of 500, 1,000, 1,500 or 2,000 ms. Subjects had to respond as fast as possible by pressing the right or left arrow key of the computer keyboard if the target was present or absent, respectively (a “TOO SLOW” feedback was displayed if response latency exceeded 5,000 ms). The screen then turned blank for 300 ms before the next trial started. Visual scenes containing 12, 24 or 48 stimuli alternated randomly across trials. There were 132 trials in total, 120 in which the target was presented with equal probability in the four search areas of the display (top right, top left, bottom right, and bottom left) and 12 “catch” trials with no target. The 120 target-present trials comprised 60 simple “Pop Out” trials where the target (circle with a bar) was easy to distinguish among distractors (plain circles), and 60 complex “Serial” trials requiring a more thorough exploration to find the target (plain circle) among distractors (circles with a bar). All subjects performed eight training trials before the experimental session.

#### Continuous Performance Task

Sustained attention in time was assessed using the CPT, a validated clinical test highly sensitive to sustained attention disorders (Riccio et al., 2002). CPT involves the rapid presentation of a long series of stimuli that mixes rare targets requiring a response (Go), and frequent distractors requiring no response (NoGo). A total of 800 pseudo-letters were presented one at a time every 1,000 ms (Figure 2D). Subjects were asked to respond quickly by releasing the space-bar of the keyboard each time the target appeared (Go trials), and to refrain from responding to all other pseudo-letters (distractors, NoGo trials). They appeared for 30 ms, followed by a 50 ms blank screen and a 30 ms mask. A 210 ms “CORRECT” or “WRONG” or “TOO SLOW” feedback, as appropriate, followed the subject response or lack thereof within the 500 ms post-target period. There were 16 blocks of 50 pseudorandomized stimuli (15 targets and 35 distractors), for a total duration of more than 13 min. All subjects performed 10 training trials before the task began.

### Tasks’ Parameters

We measured: (1) for saccades (using EyeLink^®^ DataViewer): saccadic reaction time (RT; delay from target appearance to saccade onset defined as the moment the participant’s gaze exits a 200 pixel/ ~10° square around the fixation dot), percent error (saccades in the incorrect direction or lack of response within the imparted delay), accuracy (distance between saccade endpoint and target), duration (time between saccade onset and offset), peak velocity (maximum eye speed during the saccade) and the mean pupil surface during the trial; (2) for visual search: manual RT (delay from visual scene appearance to key press), percent error (incorrect response, or lack of response within the imparted delay in target-present trials); and (3) for continuous performance: manual RT (delay from target appearance to space-bar release), percent error of Go and NoGo responses and discriminability index: *d*′ = Zhit rate (correct Go responses) − Zfalse alarm (incorrect NoGo responses) where Z follows the standard normal distribution (positive *d*′ indicates good discrimination performance).

### Individual Characteristics

We collected for all social duos the type of partner (colleague, friend, sibling, or lover) and the testing order (first or second). We also submitted all subjects (Alone and Social groups) to two personality questionnaires: (1) the French PAMA (Chalvin, 1994) using 60 questions to quantify four types of reactions to others: passive, aggressive, manipulative, and assertive; and (2) the French version of the Big Five Inventory (BFI) whose 45 items assess five personality traits (Plaisant et al., 2010): E (Extraversion, Energy, Enthusiasm), A (Altruism, Agreeableness, Affection), O (Originality, Open-Mindedness, Openness), C (Consciousness, Control, Constraint) and N (Negativity, Neuroticism, Nervousness). In addition, the duos were administered the French version (courtesy of T. Paus) of the Resistance to Peer Influence questionnaire (RPI), whose 10 questions has recently been shown to provide a measure of susceptibility to peer pressure in children, adolescents, and young adults (Steinberg and Monahan, 2007).

### Data Analysis

#### Tasks

Statistical analyses were conducted using R (RStudio, v.1.0.136). We conducted the following ANOVAs: (1) for saccades, a 2 (Condition: Social/Alone) × 2 (Protocol Difficulty: Simple/Complex) × 2 (Saccade type: Pro-/Anti-saccade) mixed design with two between-subjects factors (Condition, Difficulty) and one within-subjects factor (Saccade type); (2) for visual search, a 2 (Condition: Social/Alone) × 2 (Trials Difficulty: PopOut/Serial) mixed design with one between-subjects factor (Condition) and one within-subjects factor (Difficulty); and (3) for continuous performance, a 2 (Condition: Social/Alone) × 2 (Trials Difficulty: NoGo/Go) mixed design with one between-subjects factor (Condition) and one within-subjects factor (Difficulty). A Residual Analysis checked the Linear Model validity for each experimental design. *T*-tests were used for *post hoc* analyses. All analyses used an α error of 0.05.

#### Effect Size

The size of the social facilitation effect was evaluated using Cohen’s *d* = (MS − MA)/ SW, where MS is the mean score for the Social condition, MA is the mean score for the Alone condition, and SW the pooled within- condition standard deviation. Cohen’s rule of thumb for interpreting d values is that *d* = 0.2 represents a “small” effect size, 0.5 a “medium” effect size, and 0.8 a “large” effect size (Lakens, 2013).

#### Individual Characteristics

ANOVAs and *T*-tests, as appropriate, were used to determine whether personality differed between subjects who chose to come alone and subjects who came with a peer and, for the latter subjects, to determine whether peer presence effects changed with the type of partner they chose (friend, colleague, sibling, or lover) or with their order of testing (first or second).

### Computational Modeling of Saccade and Continuous Performance RTs

**Figure 3:**
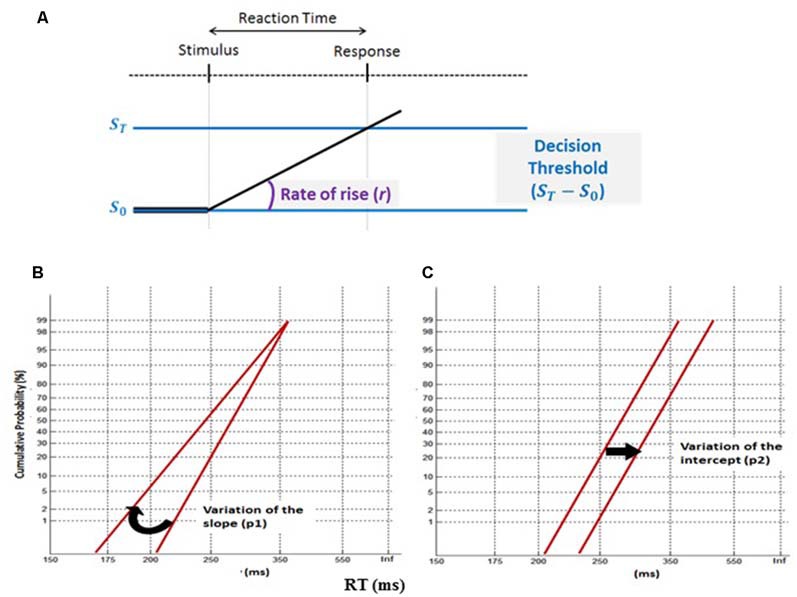
The LATER model. **(A)** This decision-making model suggests that two mechanisms cause changes of mean reaction time (RT): a tonic modulation of the decision threshold ST- S0 and a phasic change of the mean rate of rise (r). **(B)** A variation of the slope (p1) between two conditions (swiveling in regard to the time axis) reveals a change in the model decision threshold, whereas, **(C)** a variation of the intercept (p2) discloses a modulation in the rate of rise (r).

The LATER model (Figure 3), using Matlab software, was used to fit RTs recorded during correct trials in the saccade and continuous performance tasks (Carpenter and Williams, 1995; Carpenter, 2012). Socially tested subjects were compared to individually tested subjects separately for: (1) each saccade type (pro- and anti-saccades) and saccade protocol (simple and complex); and (2) the Go trials of the CPT. Thus, plotting the RT distributions under social vs. solitary testing on the same reciprobit graph could reveal which decision mechanism is most likely affected by social presence (Figures 3B,C). Two-sample Kolmogorov–Smirnov (K-S) tests were used to test differences between the distributions, and one-sample *t*-tests to identify significant changes of slope (p1) or intercept (p2) of the RT reciprobit plots.

## Results

Twelve participants were excluded (five for saccades, one for visual search, three for continuous performance) because of eye-tracker recording problems (*n* = 4), misunderstanding of the instructions (*n* = 5), or IOS scores lower than 4 (*n* = 3) denoting acquaintances rather than close relationships. The remaining socially tested subjects reached 5.6/7 (±0.2) on the IOS scale, a high score typical of close partners such as best friends (Aron et al., 1997; Myers and Hodges, 2012). Table 1 provides, for each task, the number of subjects included in the analyses. For saccades, 87.5% of the trials (35 trials per block of 40 trials, on average, for both pro-saccades and anti-saccades) were both correctly executed and properly recorded and were therefore included in the analysis presented below.

**Table 1:** Errors scores and reaction time (RT).

Error scores (%)		Condition						2 × 2 (×2) ANOVA, Condition × Difficulty (× Saccades type) or two sample *t*-test
Task	Difficulty	Social			Alone			
		*n*	mean	SEM	*n*	mean	SEM	
Saccades	Simple protocol	15	11.9	2.0	17	9.0	1.8	Condition: n.s.; Difficulty: *F*_(1,65)_ = 25, *p* < 0.001; Interaction: n.s.
	Complex protocol	19	26.4	3.0	18	20.7	2.9
Visual search	Pop-out trials	21	0.9	0.3	21	4.0	2.9	Condition: n.s.; Difficulty: *F*_(1,40)_ = 45, *p* < 0.001; Interaction: n.s.
	Serial trials	21	9.7	2.0	21	9.7	2.4
Continuous performance	NoGo trials (frequent distractors)	21	9.2	2.1	19	9.5	2.6	Condition: n.s.; Difficulty: *F*_(1,38)_ = 107.9, *p* < 0.001; Interaction: n.s.
	Go trials (rare targets)	21	37.6	3.9	19	35.6	4.5
Reaction time (ms)
Saccades	Simple protocol	15	271.5	2.2	17	285.6	1.8	Interaction Saccade type × Difficulty: *F*_(1,65)_ = 15.51, *p* < 0.001 Interaction Condition × Difficulty: *F*_(1,65)_ = 10.8, *p* = 0.002
	Complex protocol	19	320.9	1.9	18	296.9	1.6
Visual search	Pop-out trials	21	840.5	21.5	21	835.4	23.8	Condition: n.s.; Difficulty: *F*_(1,40)_ = 1,133, *p* < 0.001; Interaction: n.s.
	Serial trials	21	1,526.8	38.9	21	1579.8	38.0
Continuous performance	NoGo trials		NA			NA		
	Go trials	21	381.8	7.2	19	384.2	6.8	*t*_(38)_ = 0.24, n.s

*Group-level analyses of error scores confirmed expected difficulty effects in all three tasks, but failed to reveal any significant social influence (n.s., non-significant, i.e., *p* > 0.05). Group-level analyses of RTs outlined a difficulty effect for the visual search with no social influence for this task nor for the continuous performance. By contrast, analyses reveal two significant interactions for the saccade task: a Saccade type × Difficulty and a Condition × Difficulty interactions*.

Subjects who chose to come together with a familiar peer (Social condition) did not differ from those who chose to come alone (Alone condition) on any of the personality traits measured by the RPI, BFI and PAMA questionnaires, suggesting that this personal choice was not determined by a specific personality trait [*t*-tests, RPI: *t* = 0.41, *p* = 0.68; BFI: *t* = (−1.59, 1.44), *p*’s > 0.1 for all five personality traits; PAMA: *t* = (−0.81, 0.63), *p*’s > 0.4 for all four types of reaction to other].

The comparison between the group of subjects tested alone and the group of subjects tested in presence of a familiar partner was performed first for error scores i.e., the percentage of incorrect responses, and then for RT, in saccades, visual search and continuous performance. Additional analyses concerned kinematic parameters and pupil surface for saccades, and *d*′ values for continuous performance.

### Error Scores

For saccades, an initial ANOVA yielded no difference between pro- and anti-saccades errors (Saccade type factor: *F*_(1,68)_ = 0.7, *p* = 0.4), which were therefore pooled together. Reliable difficulty effects were observed in all three tasks, as intended: pro- and anti-saccades were more difficult when mixed than when separated (Pierce and McDowell, 2016, 2017), pop-out targets eased visual search compared to serial explorations (Khan et al., 2016), and frequent distractors (NoGo trials) were better identified than rare targets (Go trials) during the CPT (Riccio et al., 2002). Error scores showed, however, no group difference between social and solitary testing in any of the three tasks and no interaction between this and other factors, as summarized in Table 1.

### Reaction Times

**Figure 4:**
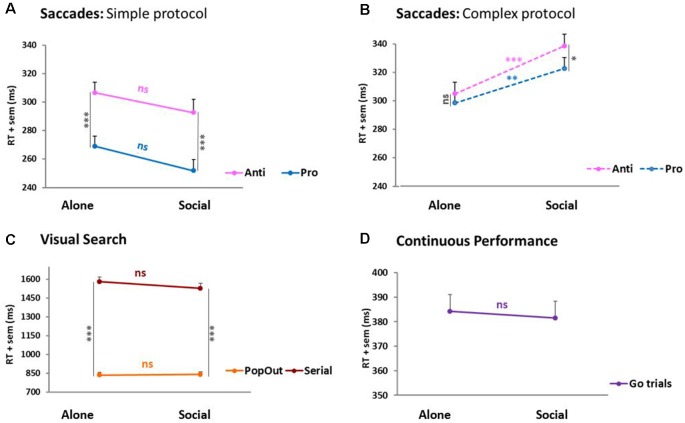
Reaction times (RTs). **(A)** Saccades: simple Protocol. **(B)** Saccades: complex protocol. **(C)** Visual search. **(D)** Continuous performance. In the simple protocol where pro- and anti-saccades were tested separately, pro-saccades RTs were ~40 ms shorter than anti-saccades RTs (***p ≤ 0.005, paired *t*-tests). In the complex protocol, where pro- and anti-saccades were mixed pseudo-randomly, this difference across saccade types was null (ns) or marginal (*p = 0.054). However, the saccades revealed a significant social influence at the group-level. In the simple protocol, saccades (pro- and anti- alike) were slightly (~15 ms) faster under social testing (Social) than under solitary testing (Alone), though the difference failed to reach significance (ns). By contrast, in the complex protocol, saccades (again, pro- and anti- alike) were both significantly slower (~20–30 ms) under social than solitary testing (pro- **p = 0.02; anti- ***p = 0.007). The two other tasks failed to show any social effect. Visual search only showed a trial difficulty effect where Serial trials were significantly longer than PopOut trials (***p < 0.001).

In visual search (Figure 4C) and continuous performance (Figure 4D), RTs failed to reveal any social influence. Only the expected difficulty effect between pop-out and serial trials was observed. A different pattern emerged for saccades (Figures 4A,B). There, a 2 × 2 × 2 ANOVA, condition × saccade type × protocol difficulty, yielded two significant interactions. The first interaction is a saccade type × protocol difficulty interaction: *F*_(1,65)_ = 15.51, *p* < 0.001, reflecting the difference between the simple and complex protocols predicted based on *Pierce and McDowell’s studies* (Pierce and McDowell, 2016, 2017). Namely, it confirms that pro-saccades become slower when mixed with anti-saccades. More importantly, the second interaction is a condition × protocol difficulty interaction: *F*_(1,65)_ = 10.8, *p* = 0.002, revealing that RTs were influenced by social presence in opposite directions according to the protocol, yielding facilitation for the simple protocol vs. an inhibition for the complex protocol. This change of saccadic RTs occurred irrespective of the testing order of socially tested subjects (first vs. second: *t*-test, *p* > 0.05 for both pro- and anti-saccades’ RTs), and did not differ with the type of partner (friend, colleague, sibling, or lover: *F* = 2.22, *p* > 0.05).

This constitutes the sole social influence on performance RT observed over the three tasks. As measured by Cohen’s *D*, the effect size amounted to 0.58 and 0.50 SDs for pro- and anti-saccades, respectively, in the saccades simple protocol, and 0.80 and 0.95 SDs in the saccades complex protocol.

### Additional Analyses

**Figure 5:**
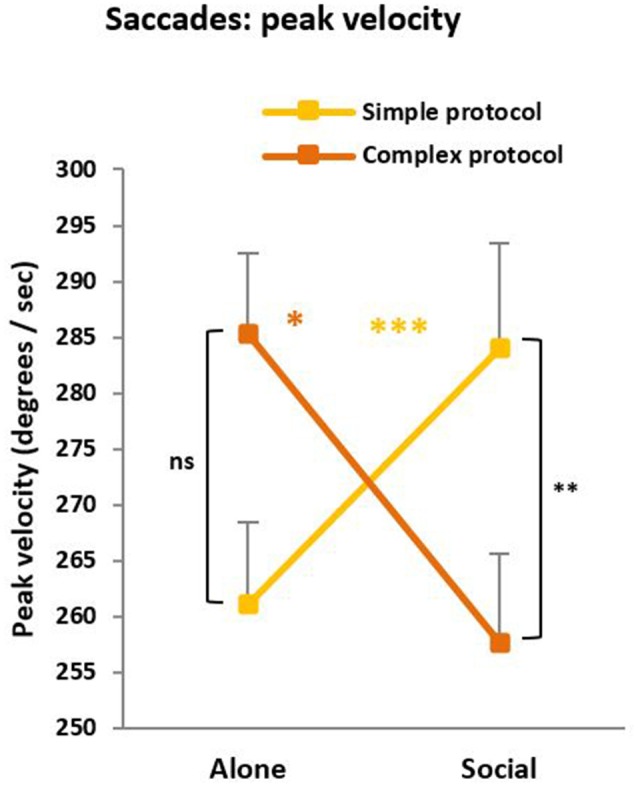
Peak velocity. Like saccade latency, peak velocity revealed a social influence whose direction depends on the protocol complexity. For the simple protocol, peak velocity was higher under social testing (Social) compared to solitary testing (Alone) suggesting a social facilitation (***p < 0.001). On the opposite, for the complex protocol, peak velocity was lower under social than solitary testing suggesting a social inhibition (*p = 0.04). Note that the difficulty effect (the difference across protocols) reached significance under social (**p = 0.01) but not solitary (^ns^*p* = 0.11) testing.

In the saccade task, saccade duration, saccade accuracy, and pupil surface were not reliably changed by the Social condition, but we found a condition × protocol interaction for the saccade peak velocity (*F*_(1,63)_ = 5.12, *p* = 0.01; Figure 5), which parallels the interaction described above for saccade RTs. For the simple protocol, peak velocity was higher under social testing compared to solitary testing, suggesting social facilitation (*p* < 0.001). On the opposite, for the complex protocol, peak velocity was lower under social than solitary testing, suggesting a social Inhibition (*p* = 0.04). Note that the difficulty effect (difference between protocols) reached significance under social (*p* = 0.01) but not solitary (*p* = 0.11) testing. Finally, in the CPT, no social influence on the *d*′ values characterizing discrimination effectiveness was found.

### Computational Modeling of Saccade and Continuous Performance RTs

**Figure 6:**
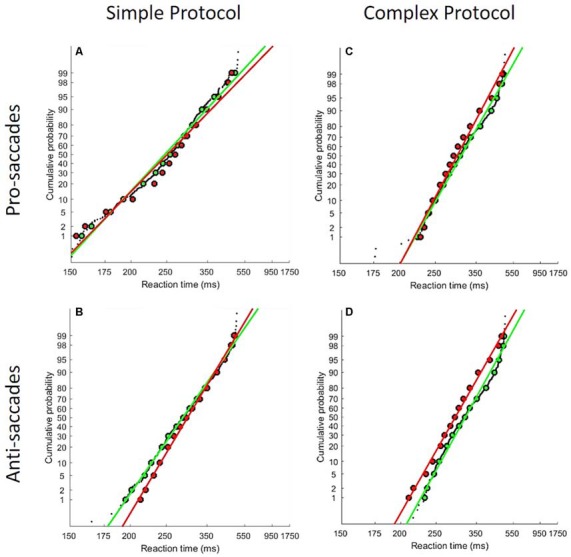
LATER model results. Reciprobit plots for each saccade protocol [simple **(A,B)** and complex **(C,D)**] and for each saccade type [pro **(A,C)** and anti **(B,D)**]. The socially tested subjects are showed in green line, and the subjects tested alone are showed in red line. As described in the main text, distributions differ significantly between the two groups of subjects in all four conditions.

One subject was excluded because too few usable trials remained after the elimination of express saccades (saccade latency < 150 ms). RT distributions did not significantly differ between the Social and Alone conditions for continuous performance (K-S, *D* = 0.02, *p* = 0.63). In contrast, a different pattern of results arose for saccade RT distributions: Figure 6 illustrates reciprobit plots of RT distributions, separately for each saccade protocol simple/complex, and each saccade type pro-/anti-saccades [all K-S comparisons were significant: (Figure 6A) Simple/Pro- *D* = 0.33, *p* < 0.001; (Figure 6B) Simple/Anti- *D* = 0.1, *p* = 0.004; (Figure 6C) Complex/Pro- *D* = 0.15, *p* < 0.001; (Figure 6D) Complex/Anti- *D* = 0.23, *p* < 0.001]. For the simple protocol (pro- and anti-saccades), differences emerged for neither p1 (Simple/Pro- *t* = −1.66, *p* = 0.11; Simple/anti- *t* = −0.76, *p* = 0.45), nor p2 (Simple/Pro- *t* = −1.32, *p* = 0.2; Simple/anti- *t* = −0.3, *p* = 0.77). By contrast, for the complex protocol, group-level analyses of p1 and p2 showed a significant difference of the intercept (p2) between the Social and Alone conditions for pro- and anti-saccades (*t-tests*, Complex/Pro- *t* = −2.4, *p* = 0.02; Complex/anti- *t* = −2.55, *p* = 0.02), with no change of the slope (p1; Complex/Pro- *t* = −1.75, *p* = 0.09; Complex/anti- *t* = −1.39, *p* = 0.18).

## Discussion

The present study compared eye movements and attention under solitary vs. social testing. Socially tested subjects were not dyads of strangers as typical in social psychology, but duos of familiar partners, a situation more representative of real-life conditions at school or at work. Therefore the results and conclusions below cannot be generalized to unknown peers. It seems reasonable, however, in light of the vast social psychology literature reporting social facilitation in presence of strangers (Bond and Titus, 1983; Guerin, 2010), to think that unknown partners would yield similar changes, though possibly of a lesser magnitude (Wechkin, 1970; Fareri et al., 2012; Herman, 2015).

### Confirmation of a Social Presence Effect on Eye Movements

The results showed a social presence effect on saccades performance for RTs, but not for errors, which interacted with task difficulty. Pro-and anti-saccades were both inhibited when pseudorandomly mixed (complex protocol), and both facilitated when performed separately (simple protocol). Effect sizes were large, with Cohen’s *d* values ranging from 0.50 to 0.95. The RT difference found between the two saccades types in the simple protocol confirms the fact that pro-saccades can be automatic saccades when executed separately, whereas anti-saccades are voluntary saccades involving more complex decision and planning processes (Gaymard, 2012; Pierce and McDowell, 2016; Coe and Munoz, 2017). Yet, anti-saccades, when executed separately, did not reach a level of difficulty sufficient to trigger the switch towards social inhibition classically described in the psychology literature for difficult tasks. In contrast, by randomly mixing them with anti-saccades in the complex protocol, pro-saccades rely on voluntary decision-making processes, and their RTs approach those of anti-saccades (Pierce and McDowell, 2016, 2017). In this case, both pro-and anti-saccades became difficult enough to produce the “canonical” switch towards social inhibition.

A social facilitation of both pro- and anti-saccades akin to that observed here in the simple protocol was reported before by McFall et al. (2009) in subjects who also performed pro- and anti-saccades in two separate blocks (of 74 trials each) and who were led to believe that their performance will subsequently be evaluated by the experimenter. The present study corroborates these earlier findings and extend them by showing that a social presence can facilitate eye movements even when it is not evaluative. In 2017, Oliva et al. (2017) tested the effect of the presence of 1–7 co-actors using a saccade protocol derived from Antoniades et al. 2013; subjects perform three blocks of 40 anti-saccades preceded and followed by a block of 40 pro-saccades) and found a social inhibition of anti-saccades in the presence of two or more co-actors. Thus, the present and earlier studies converge to emphasize the influence of social presence on saccadic RTs while suggesting that, across studies, the direction of the change varies with the specific protocol.

The consensus in social psychology is that the presence of others enhances the probability to emit prepotent or dominant responses, which are likely to be correct on simple tasks, thus leading to social facilitation, but incorrect on difficult tasks, thus leading to social inhibition (Zajonc, 1965; Harkins, 2006). The present findings show that this principle applies to whole tasks, and not to individual trials within a task, as we had initially reasoned. Rather, as proposed earlier by Bond (1982), the impairment found on complex items such as atypical anti-saccades can be eliminated when these items are embedded in an easy task and performance on simple items such as pro-saccades suffers when they are embedded in a complex task.

Analyses of saccade kinematic parameters revealed the same type of social effect and of interaction with task difficulty on peak velocity, but not on duration and accuracy. This highlights that, not only the speed to initiate the saccade is enhanced or impaired by the social presence, but also the driving force moving the eye toward the target. Kinematic parameters have been previously investigated only in visual search by Liu and Yu (2017) who, notably, also found a social effect on eye velocity.

Finally, we found no social effect on pupil size during the oculomotor tasks. Pupil size has been shown to be an indicator of arousal (Murphy et al., 2014). Contrary to our study, Liu and Yu (2017) did find a significant increase in pupil diameter in the social context during a visual search task, irrespective of the task complexity, supporting *Zajonc’s vigilance theory*. However, the present negative result must be tempered as our pupil size measures were cumulated across the whole trial, which prevented us from highlighting a temporally-specific pupil response to e.g., the target appearance or the saccadic response.

The present study thus provides the first evidence of a social modulation of saccades induced by the simple presence of a familiar peer and modulated by task difficulty, thereby strengthening a growing body of evidence showing social modulations of eye movements (McFall et al., 2009; Strukelj et al., 2012; Yu and Wu, 2015; Claypoole and Szalma, 2017; Liu and Yu, 2017; Oliva et al., 2017). This could have an important impact on real-life applications, and particularly in high stake professional activities. For example, in a test conducted by the US Transportation Security Administration, agents in charge of luggage safety checking missed 95% of targets (weapons, explosives; CNN, 2015; Claypoole and Szalma, 2017). Adding a social presence could be a low-cost and low-constraint solution to increase performance in this real-life activity, and could extend to several others (lifeguarding, military environment, manufacturing industries, cockpit monitoring…). Indeed, because the social effect is based on the individual’s belief that another person is looking (Risko and Kingstone, 2011; Richardson et al., 2012; Nakata and Kawai, 2017), it can be induced by the physical presence of a colleague or simply by its inferred but non-physical presence (cameras, avatar, photography…).

### Social Presence Effects Depend on Task Difficulty

As discussed in the previous paragraph, there was a systematic profile of social effect for both pro- and anti-saccades: facilitation in the simple protocol, and inhibition in the complex one. Comparatively, visual search and continuous performance showed no social effect on any parameter (error, manual RT, and continuous performance *d*′), a finding which contrasts with earlier reports of social facilitation and inhibition of visual search (Liu and Yu, 2017) and of social facilitation of continuous performance (Claypoole and Szalma, 2017). The present lack of social influence could indicate that the specific protocols used here for these two attentional tasks are neither as simple as our simple saccade protocol, nor as difficult as our complex saccade protocol, falling into an intermediate level of difficulty that might be easy for some individuals, but challenging to others, hence the lack of group difference.

Interindividual variability of social facilitation has been observed since Triplett’s very first study, which actually reported facilitation in half of the subjects (20/40), and no effect (10/40), or an inhibition (10/40) in the remaining subjects (Triplett, 1898; Stroebe, 2012). Yet, attempts to identify the moderators other than task difficulty that could explain such variability remain rare. Uziel (2007) proposed that personality traits may affect individual susceptibility to the social presence. Neuroticism with negative apprehensiveness and low self-esteem could increase social inhibition, whereas extraversion with optimism and high self-esteem could increase social facilitation. But so far, there is little empirical evidence supporting this theory (Stein, 2009). Self-efficacy, the belief of an individual in his/her ability to perform a specific task (Lee and Bobko, 1994), might be more critical. Sanna showed that manipulating this belief (using false performance feedback during training) suffices to reverse the outcome of social presence. High self-efficacy (induced by making subjects believe they excelled during training) led to social facilitation, while low self-efficacy (induced by making subjects believe they failed during training) led to social inhibition in the very same vigilance task (Sanna, 1992). Unlike personality traits, which are lifelong characteristics, self-efficacy is situation-dependent. Its moderating influence thus makes it possible for the same individual confronted with the same level of difficulty to be sometimes socially facilitated and sometimes socially inhibited. Further studies testing *Sanna’s self-efficacy theory and Uziel’s personality traits theory* need to be conducted simultaneously in a large number of individuals in order to build models predicting social influence for each individual depending on task difficulty. Such models could help optimize performance in many domains, especially education.

### Social Presence Effects on Decision-Making Mechanisms

To explore the mechanisms underlying social facilitation, we fitted the saccade and continuous performance RTs to Carpenter’s LATER model, which postulates two different mechanisms leading to the decision to act, i.e., move the eye or hand: top-down decision threshold and bottom-up accumulation of information (Carpenter and Williams, 1995; Carpenter, 2012). The resulting reciprobit plots of RT distributions significantly differed between the Social and Alone conditions for each saccade protocol (simple/complex) and each saccade type (pro-/anti-saccades). In their princeps study of the LATER’s parameters under solitary vs. social testing, Oliva et al. (2017) found a social inhibition associated with an increase of the threshold for anti-saccades. In the present study, social inhibition (of pro- and anti-saccades in the complex protocol) was associated instead with a significant increase of the rate of rise. Together, the present and earlier studies indicate that peer presence can influence both the top-down and bottom-up attention-related processes guiding the decision to move the eyes. This suggestion is consistent with a unified attentional theory wherein subjects could react to social presence by either a vigilance change (that we view as linked with the LATER’s threshold parameter) in line with *Zajonc’s vigilance theory* or a modulation of selective attention (that we view as linked with the LATER’s rate of rise parameter), in line with Baron’s distraction theory. Future studies are needed to test this proposal in a within-subject design, with a large number of subjects, each tested over a large number of trials, at least 120 per saccade type, as recommended by Antoniades et al. (2013). Additional computational models could also be considered to complement the first analyses presented in this report, and to better understand the mechanisms involved in social facilitation. For example, the LATEST, a derivative of LATER, could be used for visual search, since this model can both predict where and when decision making takes place (Tatler et al., 2017).

## Conclusion

The present study confirms the existence of a social presence effect on eye movements. It also demonstrates that the direction of the social effect—facilitation or inhibition—does not depend on the saccade type (automatic pro-saccades vs. voluntary anti-saccades) but on the task difficulty (successive vs. mixed protocol). Among the two LATER model mechanisms, the peer presence effect on saccades was associated with an increase in the rate of rise. Finally, the two attentional tasks (visual search and continuous performance) failed to show a significant group effect, perhaps because the specific protocols used here fall into intermediate levels of difficulty which maximize the interindividual variability of social presence effects.

## Data Availability Statement

All datasets analyzed for this study are included in the article. Original data are available upon request to the corresponding authors.

## Ethics Statement

The studies involving human participants were reviewed and approved by Ethics Committee of Inserm (IRB#00003888). The participants provided their written informed consent to participate in this study.

## Author Contributions

DP and MM designed the experiment. LT and JF-V programmed the experiment and collected the data. LT, JF-V, DP, and MM analyzed the data. LT, DP, and MM wrote the article.

## Conflict of Interest

The authors declare that the research was conducted in the absence of any commercial or financial relationships that could be construed as a potential conflict of interest.
